# Selenoprotein W redox-regulated Ca^2+^ channels correlate with selenium deficiency-induced muscles Ca^2+^ leak

**DOI:** 10.18632/oncotarget.11459

**Published:** 2016-08-20

**Authors:** Haidong Yao, Ruifeng Fan, Xia Zhao, Wenchao Zhao, Wei Liu, Jie Yang, Hamid Sattar, Jinxin Zhao, Ziwei Zhang, Shiwen Xu

**Affiliations:** ^1^ Department of Veterinary Medicine, Northeast Agricultural University, Harbin, P. R. China; ^2^ The Key Laboratory of Myocardial Ischemia, Harbin Medical University, Ministry of Education, Heilongjiang, P. R. China

**Keywords:** selenium deficiency, Ca^2+^ leak, selenoprotein W, Ca^2+^ channels, redox regulation

## Abstract

Selenium (Se) deficiency induces Ca^2+^ leak and calcification in mammal skeletal muscles; however, the exact mechanism is still unclear. In the present study, both Se-deficient chicken muscle models and selenoprotein W (SelW) gene knockdown myoblast and embryo models were used to study the mechanism. The results showed that Se deficiency-induced typical muscular injuries accompanied with Ca^2+^ leak and oxidative stress (*P* < 0.05) injured the ultrastructure of the sarcoplasmic reticulum (SR) and mitochondria; decreased the levels of the Ca^2+^ channels, SERCA, SLC8A, CACNA1S, ORAI1, STIM1, TRPC1, and TRPC3 (*P* < 0.05); and increased the levels of Ca^2+^ channel PMCA (*P* < 0.05). Similarly, SelW knockdown also induced Ca^2+^ leak from the SR and cytoplasm; increased mitochondrial Ca^2+^ levels and oxidative stress; injured SR and mitochondrial ultrastructure; decreased levels of SLC8A, CACNA1S, ORA1, TRPC1, and TRPC3; and caused abnormal activities of Ca^2+^ channels in response to inhibitors in myoblasts and chicken embryos. Thus, both Se deficiency and SelW knockdown induced Ca^2+^ leak, oxidative stress, and Ca^2+^ channel reduction. In addition, Ca^2+^ levels and the expression of the Ca^2+^ channels, RyR1, SERCA, CACNA1S, TRPC1, and TRPC3 were recovered to normal levels by N-acetyl-L-cysteine (NAC) treatment compared with SelW knockdown cells. Thus, with regard to the decreased Ca^2+^ channels, SelW knockdown closely correlated Se deficiency with Ca^2+^ leak in muscles. The redox regulation role of SelW is crucial in Se deficiency-induced Ca^2+^ leak in muscles.

## INTRODUCTION

Muscular dystrophy, such as white muscle disease (WMD) in animals and Keshan disease in humans [1], is a classical selenium (Se)/vitamin E deficiency disease [2, 3]. Se deficiency either induces typical clinical and pathological changes or may cause various pathological responses at the molecular level of muscles [4]. Many attempts have been made to elucidate the mechanism of these disorders from different angles, including oxidative stress, apoptosis, inflammation response, disordered selenoproteins, or disrupted calcium (Ca^2+^) signaling [2, 3, 5]. However, the initial molecular mechanism of Se deficiency-related muscle injuries still remains unclear.

Ca^2+^ plays important roles in the contraction, signal transduction, and enzyme active site in skeletal muscles [6]. In skeletal muscles, the influx and efflux of Ca^2+^ is regulated by several types of Ca^2+^ channels, including intracellular Ca^2+^-release channel: ryanodine receptor channel (RyR1, RyR3) and Ca^2+^ pump channel (SERCA); extracellular Ca^2+^-entry channels: L-type voltage-dependent Ca^2+^ channel dihydropyridine receptors (DHPR or CACNA1S), transient receptor potential channels (TRPC1, TRPC3, and others), Ca^2+^-release-activated Ca^2+^ current channels (CRAC); and extracellular Ca^2+^-entry balancing channels: Na^+^/Ca^2+^ exchanger (NCX), plasma membrane Ca^2+^-ATPases (PMCA) and others [7]. The activities of these Ca^2+^ channels are closely related to the biological function of skeletal muscles and diseases of the muscles. Early observations demonstrated that the sarcoplasmic reticulum (SR) of Se deficiency muscle is defective in Ca^2+^ sequestration, resulting in extensive calcification of the muscle tissues [3]. Ca^2+^ disruption or Ca^2+^ channel inactivation in different types of cells was also correlated with disordered selenoproteins (SelN, SelT, SelK or SelM) [8–12], the executors of the biological function of Se. These pathological and molecular changes in muscles connect Se deficiency-related muscle injuries with Ca^2+^ signal disruption. However, the effect of Se deficiency on Ca^2+^ homeostasis has been less studied, and the mechanism of Ca^2+^ disruption in Se deficiency muscles still remains unclear. Although Se deficiency diseases models have been established in mice, pig, cow, lamb, and chicken [2, 3, 13, 14], whether the typical pathological changes, such as Ca^2+^ leak and calcification, in Se deficiency diseases can be replicated using currently available practical diets has yet to be determined.

Selenoprotein W (SelW) is the first selenoprotein linked to Se deficiency-related muscular disorders [15]. SelW is less abundant in the muscles of WMD animals, and WMD animals have demonstrated defective Ca^2+^ levels in the SR and calcification in muscles [3]. Therefore, SelW may have a close relationship with Ca^2+^ signals and Se deficiency-related muscle damage. However, to the extent of our knowledge, there is still no direct report on the role of SelW in Ca^2+^ regulation or the link between SelW and Ca^2+^ disorder in Se deficiency muscles. Thus, we conducted the present study to determine 1) whether and how Se deficiency influences Ca^2+^ signals in chicken muscles and 2) whether SelW plays a role in the regulation of Ca^2+^ homeostasis *in vitro* and/or *in vivo*. The present study provides insights into the effects of Se and SelW on Ca^2+^ signaling and the physiological role of SelW in muscular dystrophy.

## RESULTS

### Se deficiency-induced Ca^2+^ leak in chicken muscles

**Figure 1:**
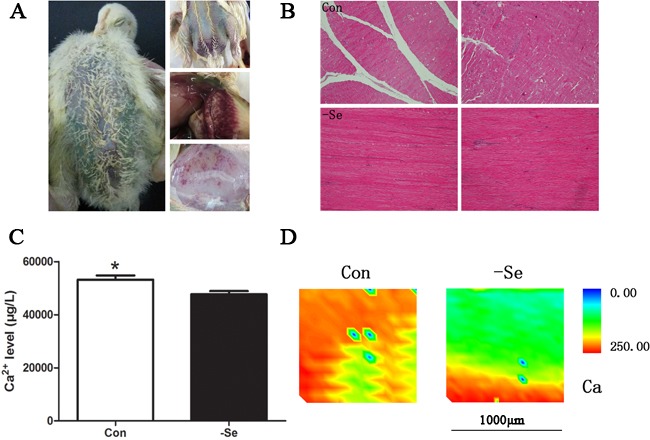
Se-deficiency chicken muscles **A.** The typical exudative diathesis and extravasated blood in muscles; **B.** HE staining for muscles, 100×; **C.** the Ca^2+^levels in chicken muscles; **D.** Ca^2+^ image detected by SRμ-XRF technology where red showed the high Ca^2+^ levels and blue showed low Ca^2+^ levels. Ca^2+^ levels in chicken muscles were assessed using Student's *t*-test. The data are expressed as the means ± SD, *n* = 5. * shows the significant difference, *P* < 0.05.

In the present study, we treated the broiler chickens with a Se-deficiency diet for 25-30 days. During that time, the chickens demonstrated typical exudative diathesis, dyskinesia, extravasated blood in muscles, or even death (Figure 1A). To ensure that the Se deficiency models were successfully established, we also examined Se levels in muscles (unpublished data). At the molecular level, we found disordered selenoproteins, oxidative stress, and apoptosis [2, 16]. In agreement with previous reports, Se deficiency-related chicken disease can be successfully established by simply feeding a Se-deficient diet. As indicated by a previous study, Ca^2+^ leak and muscle calcification were always accompanied with muscle injuries in lamb [3]. In the present study, we detected total Ca^2+^ levels in the muscles using ICP-MS technology (Figure 1C) and found that the total Ca^2+^ levels were decreased by Se deficiency (*P* < 0.05). In addition, the distribution of Ca^2+^ in skeletal muscles by SRμ-XRF demonstrated lower Ca^2+^ levels in muscle sections (Figure 1D), which further verified Ca^2+^ leakage in Se-deficient muscles. No obvious calcification was observed by HE staining in either group (Figure 1B).

### Ca^2+^ levels in SelW deficiency chicken myoblasts

**Figure 2:**
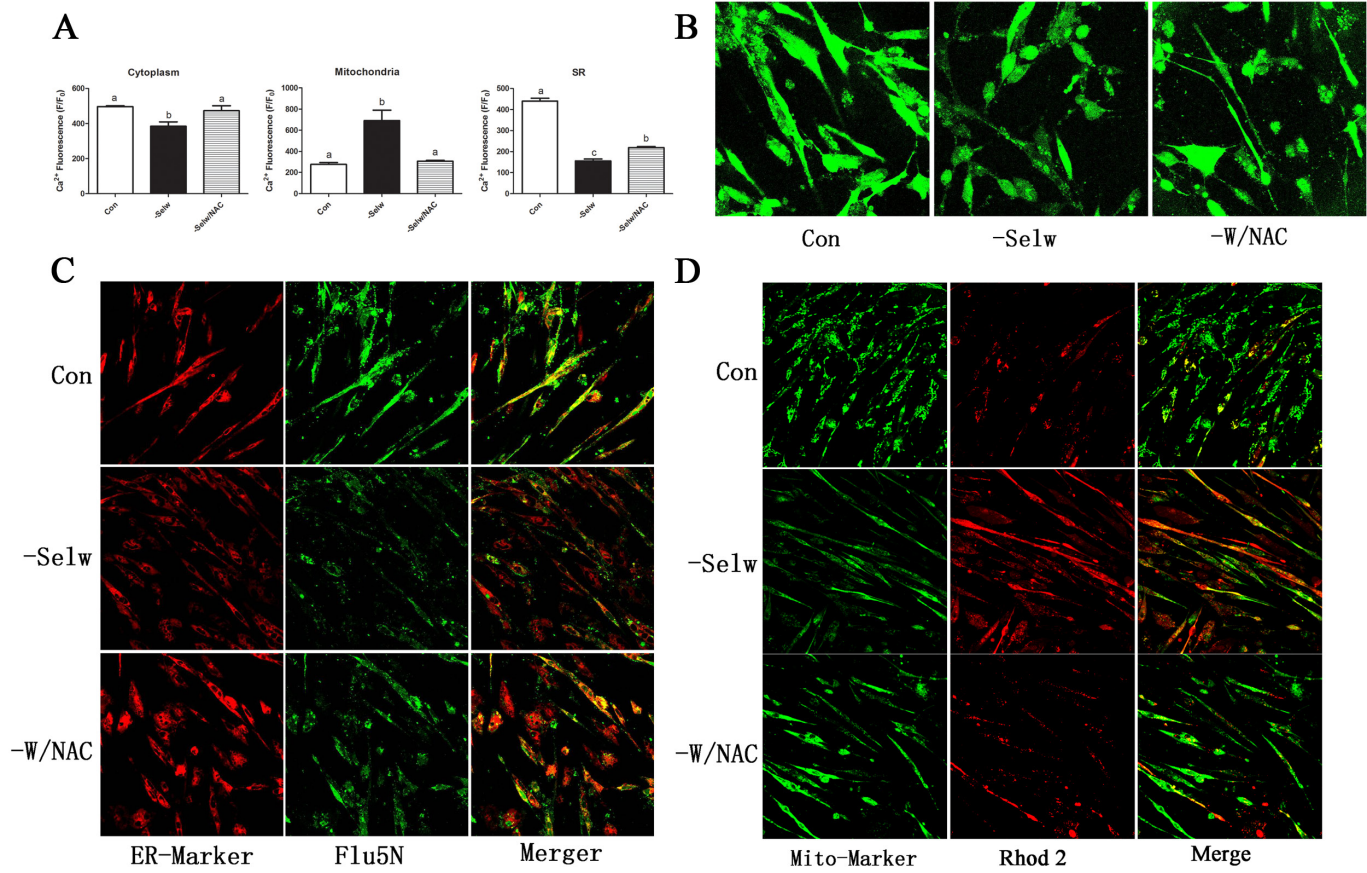
Ca^**2+**^ levels detection **A.** Ca^2+^ levels in myoblast; **B.** Ca^2+^ levels in cytoplasm detected by Fluo3 fluorescence; **C.** Ca^2+^ levels in SR detected by Fluo5N fluorescence; **D.** Ca^2+^ levels in mitochondria detected by Rhod2 fluorescence. ER-Marker fluorescently labeled SR while Mito-Marker fluorescently labeled mitochondria. After the cell fluorescence was equilibrated and stabilized, the fluorescence was measured with a confocal laser scanning microscope using a 40× oil lens and analyzed using the Olympus Fluoview Ver. 2.0a Viewer software. The fluorescence intensity levels are presented relative to baseline and shown as F/F_0_. At least 8-13 cells were analyzed. Different lowercase letters show the significant difference, *P* < 0.05.

In the present study, we silenced the expression of SelW by siRNA for 48 h and used different Ca^2+^ indicators to detect the Ca^2+^ levels in myoblasts. To verify the knockdown efficiency and exclude off-target effects, we previously constructed three different target siRNAs and two negatives [17], and the SelW expressions were decreased more than 77%. In the present study, we used these validated siRNAs to treat myoblasts. These results demonstrated that SelW deficiency decreased the level of Ca^2+^ in cytoplasm (Figure 2A, Figure 2B) and SR (Figure 2A, Figure 2C) (*P* < 0.05) and increased Ca^2+^ levels in mitochondria (Figure 2A, Figure 2D) (*P* < 0.05). These data also support the hypothesis that SelW-related muscular disease is defective of Ca^2+^ levels in SR [3].

Following SelW deficiency, we treated cells with NAC, a global antioxidant. In our previous study, we showed that NAC treatment decreased ROS levels and apoptosis, following the SelW deficiency and the H_2_O_2_ treatments [17]. Therefore, NAC was an efficient antioxidant in primary culture myoblasts that alleviated the oxidative injuries. NAC treatment increased cytoplasmic Ca^2+^ (Figure 2A, Figure 2B) and decreased mitochondrial Ca^2+^ (Figure 2A, Figure 2D) to normal levels (*P* < 0.05) and alleviated the Ca^2+^ release from SR (Figure 2A, Figure 2C) (*P* < 0.05). These results demonstrated that NAC alleviated the effect of SelW deficiency (*P* < 0.05) in the cytoplasm and mitochondria. However, the Ca^2+^ signal in SR is still lower than the control (*P* < 0.05). Thus, SelW may partially influence the Ca^2+^ signal by regulating the oxidative stress.

### Ca^2+^ levels in SelW knockdown chicken embryo skeletal muscles

**Figure 3:**
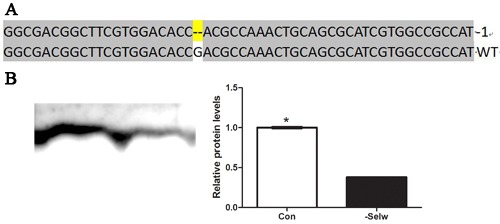
The sequence of SelW in embryo **A.** The sequences of SelW from SelW-knockdown embryo muscles. Deletions are indicated with (--); **B.** the proteins levels of SelW where β-actin was selected as the reference and the related proteins levels were assessed using Student's *t*-test. The data are expressed as the means ± SD, *n* = 5, * shows the significant difference, *P* < 0.05.

**Figure 4:**
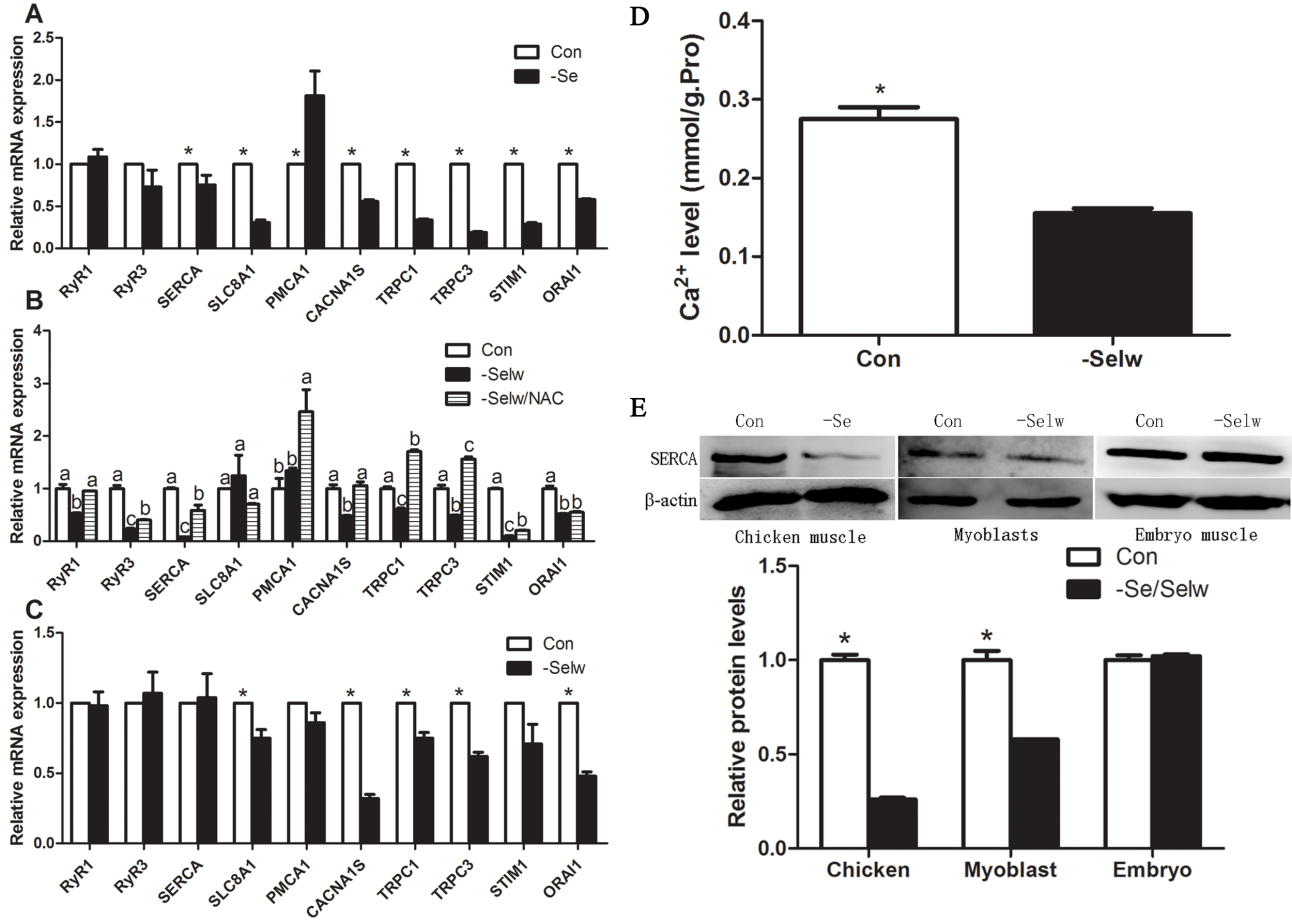
The expression levels of Ca^**2+**^ channels and Ca^**2+**^ levels in embryo muscles **A.** mRNA levels of Ca^2+^ channels in chicken muscles; **B.** mRNA levels of Ca^2+^ channels in myoblasts; **C.** mRNA levels of Ca^2+^ channels in embryo muscles; **D.** Ca^2+^ levels in embryo muscles; **E.** the protein levels of SERCA1S were detected by western blot technology and β-actin was selected as the reference. Ca^2+^ channels in chicken muscles, Ca^2+^ levels in embryo muscles, and protein levels of Ca^2+^ channels were assessed using Student's *t*-test while Ca^2+^ channels in myoblasts were assessed using one-way ANOVA. The data are expressed as the means ± SD, *n* = 5, * and different lowercase letters shows the significant difference, *P* < 0.05.

To further identify the Ca^2+^ regulatory role of SelW on Ca^2+^ and the role of Se deficiency-related muscles on Ca^2+^ disruption, it was necessary to determine the role *in vitro*. In the present study, we injected the Cas9 SelW plasmid into a Stage X chicken embryo, collected the skeletal muscles at E7d embryo, and sequenced the transcript of SelW. The results (Figure 3A) demonstrated that in the CDS region of SelW, there is one deleted nucleotide. In addition, the knockdown efficiency (Figure 3B) was validated by western blot. The protein levels were decreased by more than 62% (*P* < 0.05). Therefore, the SelW knockdown embryo models were established by Cas9 technology. Then, we screened the SelW knockdown models to check the levels of Ca^2+^ and other biomarkers. The results (Figure 4D) showed that, similar to myoblasts, SelW deficiency led the decrease of total Ca^2+^ levels in E7d embryo skeletal muscles (*P* < 0.05). The *in vitro* experiment further verified the role of SelW in the regulation of Ca^2+^ and that SelW deficiency was closely related to Ca^2+^ leak in muscles.

### Se deficiency influence Ca^2+^ channels expression in chicken muscles

In the present study, we detected 10 Ca^2+^ channels: including RyR1, RyR3, SERCA1s, TRPC1, TRPC3, CACNA1S, CRAC (STIM1, ORAI1), NCX (SLC8A1), and PMCA1 (Figure 4A and Figure 4E). Se deficiency induced the decrease of SERCA, SLC8A, CACNA1S, ORAI1, STIM1, TRPC1, and TRPC3 (*P* < 0.05) and the increase of PMCA (*P* < 0.05), but it did not influence RyR1 or RyR3 (*P* > 0.05). All extracellular Ca^2+^-entry channels were decreased, while intracellular Ca^2+^-release channels were not influenced. Lower levels of extracellular Ca^2+^-entry channels decrease the Ca^2+^ transporting activities, but higher PMCA, lower SERCA, and unchanged RyR1 and RyR3 keep Ca^2+^ leak from SR. Thus, taking into account the lower levels of total muscle Ca^2+^ levels, it is reasonable to hypothesize that Se deficiency induced Ca^2+^ leak in muscles by influencing Ca^2+^ channels.

### SelW deficiency influenced the expression of Ca^2+^channels in chicken myoblasts

In chicken myoblasts, SelW deficiency (Figure 4B, Figure 4E) decreased the levels of RyR1, RyR3, CACNA1S, SERCA1, TRPC1, TRPC3, STIM1, SERCA1, and ORAI1 (*P* < 0.05) but did not influence SLC8A1 or PMCA Ca^2+^ channels (*P* > 0.05). Thus, the effect of SelW deficiency on Ca^2+^ channels (CACNA1S, SERCA, TRPC1, TRPC3, STIM1, and ORAI1) was the same as Se deficiency in muscles.

**Figure 5:**
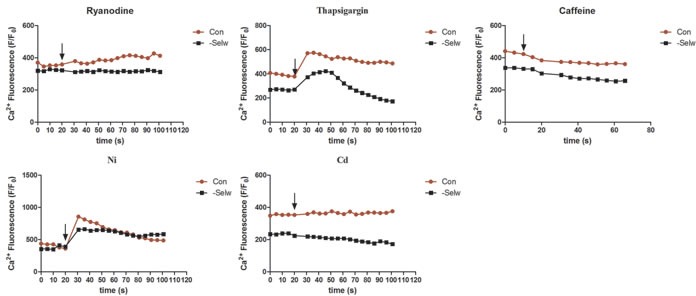
The Ca^**2+**^ levels following the treatment with inhibitors and activators After the cell fluorescence was equilibrated and stabilized, Ni, Ryanodine, Cd, Caffeine, Thapsigargin were used to treat the cells, and the fluorescence changes measured. At least 8-13 cells were analyzed, *n* = 5.

To identify the effect of SelW on Ca^2+^ channels, we also treated myoblasts with the specific Ca^2+^ channel inhibitors (ryanodine for RyR1; thapsigargin for SERCA; Cd for CACNA1S; and Ni for SLC8A1) and activator (caffeine for RyR1). After the cell fluorescence was equilibrated and stabilized, we treated the cells with different inhibitors and activators separately and observed the cellular response. The results (Figure 5) demonstrated that the response of cells to caffeine was not significantly different than control and siRNA cells; however, the effects of ryanodine, Ni, thapsigargin, and Cd on cells were different. When treated with ryanodine, control cells showed a sustained increase in Ca^2+^ signal, while in siRNA cells, the Ca^2+^ levels remained stable, Therefore, the inhibiting effect of ryanodine on control cells was more significant than siRNA cells. Both groups of cells showed rapid responses to thapsigargin and then reduced to a lower level. However, siRNA cells recovered to the normal levels earlier (more than 25 s) than control and were even reduced to a lower level than normal levels. Therefore, the sensitivity of SERCA to thapsigargin is increased in siRNA SelW. Ni treatment induced a higher and more rapid response in control cells with shorter duration than siRNA cells. Ni treatment maintained a higher Ca^2+^ level in siRNA cells. In siRNA cells, the NCX (SLC8A1) channels showed a sustained response to Ni. Cd treatment decreased the Ca^2+^ levels in siRNA cells, but did not influence Ca^2+^ levels in control cells. SelW deficiency increased the sensitivity of cells to Cd treatment. Thus, excepting the inhibitory effect of SelW deficiency on the expression of Ca^2+^ channels, SelW deficiency also influenced the activities of RyR1, SERCA, CACNA1S, and SLC8A1 in myoblasts.

In addition, after the treatment of NAC, the Ca^2+^ channels were also influenced by SelW deficiency. NAC treatment increased RyR1, CACNA1S, TRPC1, and TRPC3 to a normal or higher level (Figure 4B) (*P* < 0.05) and alleviated the effect of SelW deficiency on RyR3, SERCA, and STIM1 (*P* < 0.05). Therefore, the effect of SelW on Ca^2+^ channels was partially dependent on the redox-regulation role.

### SelW deficiency influenced the expression of Ca^2+^ channels in embryo muscles

Similar to the Se deficiency muscles, we also detected 10 Ca^2+^ channels in embryo muscles. The results (Figure 4C and Figure 4E) showed that SelW knockdown decreased the levels of SLC8A, CACA1S, ORA1, TRPC1, and TRPC3 (*P* < 0.05), but other Ca^2+^ channels were not influenced (*P* > 0.05). It was observed that SelW influenced less Ca^2+^ channels than Se deficiency, but the effect of SelW knockdown on Ca^2+^ channels (SLC8A1, CACNA1S, TRPC1, TRPC3, and ORAI1) was similar. In the *in vivo* experiment, SelW primarily decreased the levels of extracellular Ca^2+^-entry channels, similar to Se deficiency.

### Antioxidative enzyme activity and oxidative injuries

**Figure 6:**
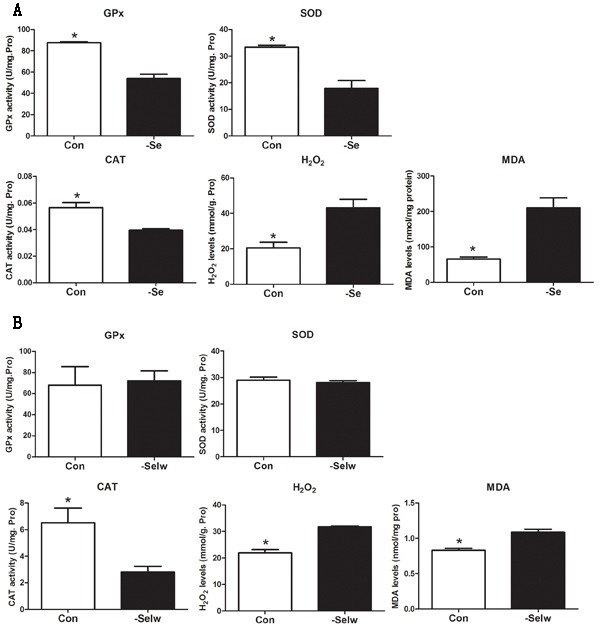
Enzymes activity detection **A.** Enzymes activities in chicken muscles; **B.** Enzymes activities in embryos. Enzymes activities were assessed using Student's *t*-test. The data are expressed as the means ± SD, *n* = 5. * shows the significant difference, *P* < 0.05.

To examine the redox state in chicken and embryo, we detected the activities of antioxidative enzymes GPx, SOD, and CAT and measured H_2_O_2_ and MDA levels. The results (Figure 6A) showed that Se deficiency decreased the activities of GPx, SOD, and CAT while increasing H_2_O_2_ and MDA levels in chicken muscles (*P* < 0.05). However, in embryo muscle (Figure 6B), SelW knockdown decreased the activity of CAT only (*P* < 0.05), not GPx and SOD (*P* > 0.05). The H_2_O_2_ and MDA levels were increased (*P* < 0.05). In our previous study, we also detected the ROS concentration, MDA levels, and antioxidative enzymes in myoblasts, which showed oxidative stress in SelW-silenced myoblasts [16, 17]. Therefore, both Se deficiency and SelW knockdown induced oxidative stress.

### Ultrastructure detected by electron microscopy

**Figure 7:**
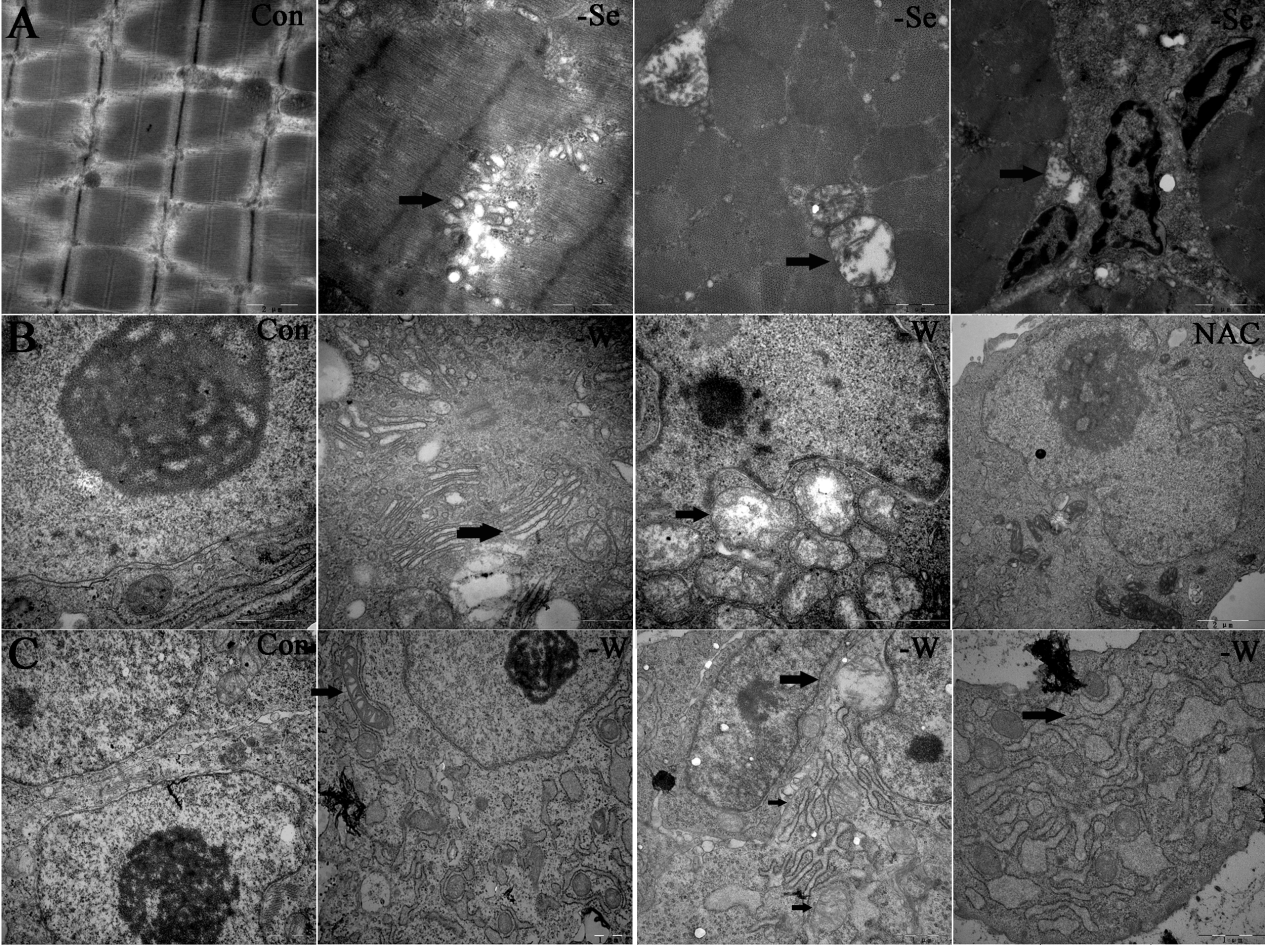
Ultrastructure detected by electron microscopy **A.** Ultrastructure for chicken muscles; **B.** ultrastructure for myoblasts; and **C.** ultrastructure for embryo muscles. Black arrows show the mitochondrial swelling, vacuolization, and disruption of mitochondrial cristae, typical apoptosis structure, the swollen and dilated SR. 15000-40000 ×magnification, -W is -SelW, NAC is -SelW/NAC.

In the present study, we detected the ultrastructure of chicken muscles, myoblast, and embryo muscles. The results (Figure 7) demonstrate that Se and SelW deficiencies primarily induced swelling and dilation in both SR and mitochondria, vacuolization, and disruption of the mitochondrial cristae, which has been shown to be closely related to Ca^2+^ leak and oxidative stress [18]. Myoblasts were also treated with NAC, and it was found that NAC alleviated the histopathological alterations in SR and mitochondria.

### Mitochondrial membrane potential in myoblasts

**Figure 8:**
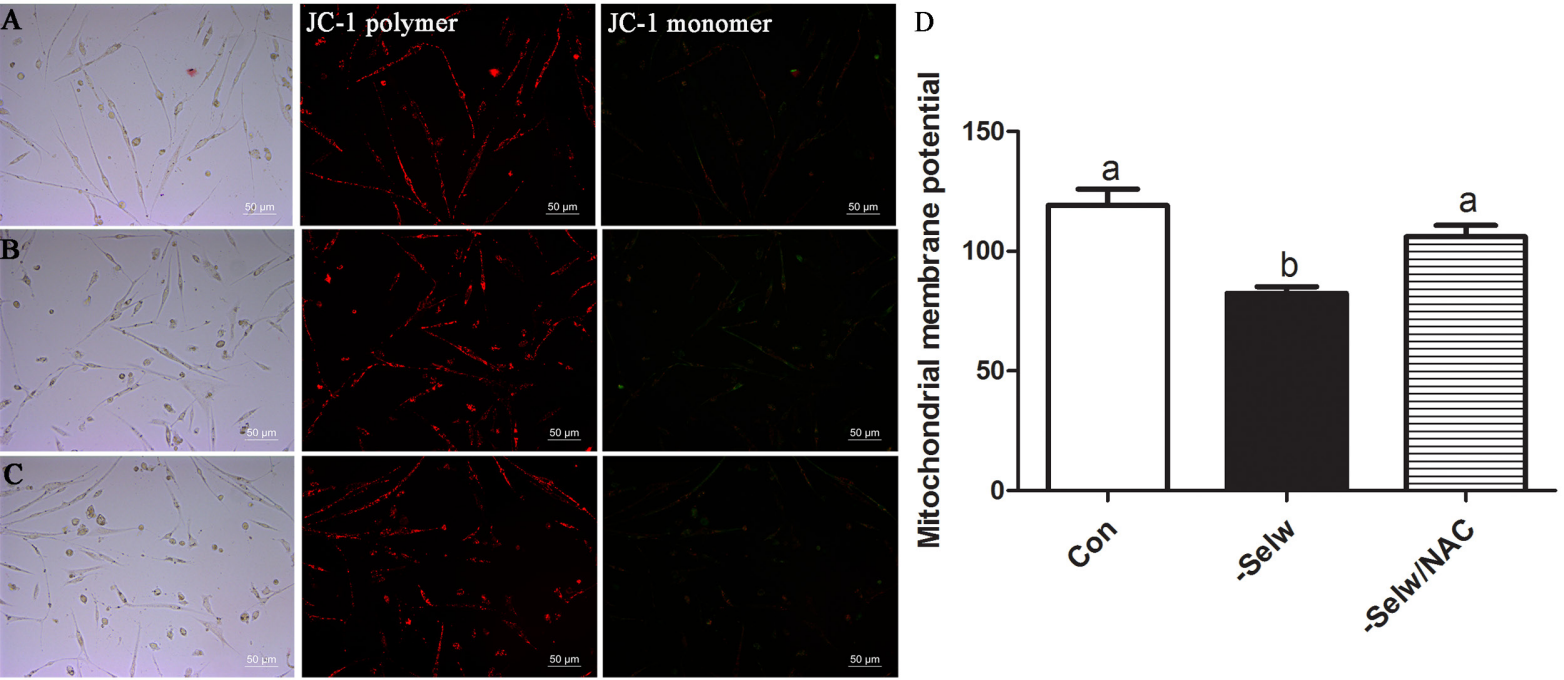
Mitochondrial membrane potentials (ΔΨm) **A.** ΔΨm of Con; **B.** ΔΨm of -SelW; **C.** ΔΨm of -SelW/NAC. (A-C) ΔΨm monitored by fluorescent microscope: red fluorescence indicates the aggregates, JC-1, while green fluorescence indicates the monomer; **D.** ΔΨm monitored by Multimode Plate Readers. ΔΨm in myoblasts were assessed using one-way ANOVA. The data are expressed as the means ± SD, *n* = 5. Different lowercase letters show the significant difference, *P* < 0.05.

Mitochondrial membrane potential (ΔΨm) is always influenced by substantial amounts of cytosolic Ca^2+^ and mitochondrial Ca^2+^. In the present study, we measured ΔΨm using two different technologies. Both results (Figure 8) showed that SelW deficiency deceased ΔΨm (*P* < 0.05), while the NAC treatment alleviated the ΔΨm (*P* < 0.05). Similar to previous studies, which demonstrated that excessive Ca^2+^ accumulation in mitochondria induced mitochondrial dysfunction and injury, ΔΨm was also influenced by the treatment of NAC [18, 19]. Therefore, the redox regulation function of SelW plays important role in the mitochondrial injuries.

## DISCUSSION

Ca^2+^ leak and calcification in skeletal muscles was reported in Se deficiency-related muscular dystrophy [3], which indicates the involvement of Ca^2+^ disorder in Se deficiency muscle injuries. However, fewer reports demonstrated the effect of Se deficiency on Ca^2+^ signal in animals, and the mechanism by which pathological changes occur. In chicken, we successfully replicated Se deficiency-related muscle disease and observed the typical pathological and molecular changes, such as oxidative stress, apoptosis, disordered selenoproteins [2, 20], SR, and mitochondrial injuries; and Ca^2+^ leak was reported only in lamb [3]. The present study supported the hypothesis that Ca^2+^ disruption was involved in the muscle disease induced by Se deficiency. To the extent of our knowledge, this is the first report to show the effect of Se deficiency on Ca^2+^ signal in chicken muscles.

Ca^2+^ signal in muscles is tightly regulated by several types of Ca^2+^ channels and other molecules [7]. The disruption of Ca^2+^ channels will influence the Ca^2+^ signal, and induce Ca^2+^ channelopathies [7, 21–25]. Several previous studies showed the direct or indirect link of Ca^2+^ leak or disruption with TRPCs and NCX [26]; SERCA, RyRs, and NCX [27, 28]; STIM1 [29]; and PMCA or SERCA [30]. However, less documentation about the effect of Se deficiency on Ca^2+^ signal in chicken muscles is available. In the present study, we selected 10 Ca^2+^ channels and hoped to screen out one or some Ca^2+^ channels related to Se deficiency muscle injuries. The results showed that Se deficiency decreased the expression of SLC8A, CACNA1S, ORAI1, STIM1, SERCA, TRPC1, and TRPC3; increased the expression of PMCA; and did not influence RyR1 and RyR3 levels. The reduced extracellular Ca^2+^-entry channels and SERCA may well explain the Ca^2+^ leak in muscles; however, Ca^2+^ leak may also influence the levels of some Ca^2+^ channels [26, 31]. Therefore, the relationship between altered Ca^2+^ channels and Ca^2+^ leak in muscles is a complex. Excepting the close relationship of TRPCs, STIM1, and SLC8A channels with Ca^2+^ leak, the most important Ca^2+^ channels involved in the regulation of SR Ca^2+^ levels are RyRs and SERCA [28]. In addition, RyRs, TRPCs and SERCA are less reported Ca^2+^ channels to be related to Se treatment [32, 33] and redox regulation activities [34, 35]. The present study concluded that the Ca^2+^ channels TRPC1, TRPC3, STIM1, SLC8A, SERCA, PMCA1, and CACNA1S are involved in Se deficiency-induced Ca^2+^ leak. Compared with previous studies, we reported two new Ca^2+^ channels, PMCA1 and CACNA1S that were related to Se deficiency and Ca^2+^ leak but found that RyRs were not involved in the Se deficiency-induced Ca^2+^ leak.

Another possible link between Se deficiency and Ca^2+^ disruption is the selenoproteins. Selenoproteins, such as SelN, SelM, SelT, SelK, SelW, and Txnrd, have either direct or indirect relation with Ca^2+^ signal and/or Ca^2+^ channels regulation [3, 8–12, 32]. Among the 19 selenoproteins decreased by Se deficiency in broiler chicken muscles [20], only Txnrd2, Txnrd3, SelK, and SelW were reported to relate to Ca^2+^ regulation. Among these, SelW has a close relationship with the biological function and injuries of muscles [3, 17, 36, 37]. In the present study, we selected SelW as the primary candidate selenoprotein. After the deficiency of SelW was induced, primary myoblasts showed reduced Ca^2+^ levels in cytoplasm and SR, but accumulation in mitochondria. Similar to the myoblast, SelW knockdown in embryo muscles also induced Ca^2+^ leak. In addition, SelW deficiency also induced typical SR and mitochondrial injuries, which were closely related to Ca^2+^ leak, oxidative stress, and disordered Ca^2+^ channels [18, 38]. Both *in vivo* and *in vitro* experiments indicated that Ca^2+^ leak and the related muscles injuries occur subsequent to SelW deficiency, which supported the idea that SelW deficiency is actively involved in the mechanism of Se deficiency-induced Ca^2+^ leak in muscles. However, by detecting the response of Ca^2+^ channels to SelW deficiency, we found that SelW deficiency also influenced the Ca^2+^ channels. In myoblasts, SelW deficiency reduced RyR1, RyR3, SERCA, CACNA1S, TRPC1, TRPC3, STIM1 and ORAI1 Ca^2+^ channels. In embryos, SelW deficiency reduced SLC8A, CACNA1S, ORA1, TRPC1, and TRPC3 Ca^2+^ channels. The effect of SelW deficiency on CACNA1S, ORA1, TRPC1, and TRPC3 was similar to the treatment of Se deficiency. Therefore, SelW may play roles in the Se deficiency-induced Ca^2+^ leak by regulating the expressions of some typical Ca^2+^ channels.

How Se and SelW deficiency influenced the Ca^2+^ signal and the Ca^2+^ channels remains an unsolved question. In the present study, we treated myoblasts with different Ca^2+^ channel inhibitors and an activator to check the response of cells. The effect of ryanodine, Ni, Cd, and thapsigargin on cells was different. SelW deficiency reduced the response of RyR1 to ryanodine, increased the sensitivity of SERCA to thapsigargin, and CACNA1S to Cd, or sustained and delayed the effect of Ni on NCX. However, caffeine does not induce any change in response in the control or siRNA cells. Therefore, the expression levels were influenced by SelW, as were the activities of some Ca^2+^ channels. As SelW is the typical antioxidative selenoprotein in myoblast, the redox regulation role may link SelW closely with these Ca^2+^ channels. In addition, oxidative stress was detected in Se deficiency chicken muscles and SelW knockdown embryo muscles and myoblasts [16, 17]. Therefore, oxidative stress closely linked SelW with Se deficiency-related injury. Following this, we treated the siRNA cells with NAC, a typical antioxidant. The results showed that NAC treatment alleviated the effect of SelW deficiency on RyR1, RyR3, SERCA, STIM1, CACNA1S, TRPC1, and TRPC3. Therefore, these Ca^2+^ channels were regulated by SelW in a redox-dependent manner. As in the Ca^2+^ channels, the Ca^2+^ levels in cytoplasm and mitochondria and the ROS levels [17] recovered to the normal levels. Even in SR, Ca^2+^ leak was alleviated by NAC treatment. In addition, the SR and mitochondria injuries induced by SelW deficiency were also alleviated by NAC treatment. Therefore, the regulation of Ca^2+^ by SelW was partially dependent on the redox regulation function of SelW. This agrees with previous studies showing that redox activities of some Ca^2+^ channels, such as SERCA, RyR1, and TRPC1 [34, 35], could be regulated by antioxidant or oxidant [18, 39]. Therefore, redox-regulated SERCA, CACNA1S, TRPC1, and TRPC3 channels by SelW may be involved in the process of Se deficiency-induced Ca^2+^ leak.

An important unanswered question that emerges from our experimentation is how SelW related Ca^2+^ leak functions with its redox regulation. One possible explanation in this context is that the SelW deficiency may decrease the antioxidative ability of myoblast [17] and muscles, and then this increased oxidative stress regulates the activation of Ca^2+^ channels such as SERCA and TRPCs, and leads to Ca^2+^ leak. In contrast, Ca^2+^ leak induced by SelW deficiency increased the accumulation of Ca^2+^ in mitochondria, and then this increased mitochondrial Ca^2+^ led to the production of reactive oxygen species [17, 34] and decreased mitochondrial membrane potential or increased mitochondrial damage [34]. Therefore, the SelW deficiency induced a vicious circle of molecular changes in muscles. By redox regulation function, SelW associated to SR and mitochondria together in the SelW deficiency-induced Ca^2+^ leak and oxidative stress.

In summary, the typical pathological changes of skeletal muscles Ca^2+^ leak can be successfully replicated by solo feeding broiler chicken with a Se-deficient diet. In this process, Se deficiency primarily decreased the extracellular Ca^2+^-entry channels and SERCA, and influenced the Ca^2+^ signals. In addition, SelW deficiency induced the Ca^2+^ leak in both *in vitro* myoblast and *in vivo* embryo muscles, and influenced the Ca^2+^ channels in embryo muscles, which indicates that SelW is involved in the process of Ca^2+^ leak induced by Se deficiency. In the present study, we screened Ca^2+^ channels: TRPC1, TRPC3, STIM1, ORA1, SLC8A, SERCA, PMCA1, and CACNA1S in chicken muscles and SLC8A, CACNA1S, ORA1, TRPC1, and TRPC3 in myoblasts and embryos that were involved in the Ca^2+^ leak. By treating the myoblasts with different Ca^2+^ channel inhibitors and NAC, we also found that redox-regulation role of SERCA, CACNA1S, TRPC1, and TRPC3 by SelW was closely related to Ca^2+^ leak. We concluded that SelW plays an important role in the Se deficiency-induced muscles Ca^2+^ leak.

## MATERIALS AND METHODS

### Birds and diets

All procedures used in this study were approved by the Institutional Animal Care and Use Committee of Northeast Agricultural University [20]. A total of 180 one-day-old male broiler chicks (Weiwei Co. Ltd., Harbin, China) were randomly divided into two groups (90 chickens per group). Over the entire experimental period, the chickens were allowed ad libitum consumption of feed and water. The chickens were maintained on either a Se-deficient diet (-Se group) containing 0.008 mg Se/kg or a sodium selenite diet (Control group) containing 0.2 mg Se/kg. Each group was separated into six pens (15 chickens each pen). Chickens were killed at 25-30 days old. Following euthanasia with sodium pentobarbital, pectoral muscles were quickly removed. The tissues were rinsed with ice-cold sterile deionized water, frozen immediately in liquid nitrogen, and stored at −80°C until needed.

### Cell culture and treatment

Primary cultures of chicken embryo-driven myoblasts were prepared as described [17]. Briefly, myoblasts were isolated from the pectoral muscle of 12-day-old chicken embryos and digested with 0.1% collagenase I (Invitrogen, Carlsbad, CA, USA). The cell suspension was washed twice and separated with Percoll (Pharmacia, Uppsala, Sweden). The myoblasts were seeded into gelatin-coated six-well culture plates at a density of 12 × 10^4^ cells/cm^2^ and were allowed to proliferate for 24 h in 5% CO_2_ at 37°C. Later, the differentiation of myoblasts was induced by replacing the proliferation medium with differentiation medium.

After the chicken myoblasts were plated in six-well plates at 70-80% confluence, the cells were transfected with 3 μL of 20 μM siRNAs and 3 μL of Lipofectamine RNAiMAX Reagent (Invitrogen) in 2 mL of Opti-MEM. After transfection for approximately 48 h, the cells were harvested for analysis. In the N-acetyl-L-cysteine (NAC) group, the cells were co-incubated with 2.5 mM NAC for 6 h after transfection and then harvested for analysis.

### Cas9 plasmid construction and injection in chicken embryo

CRISPR/Cas9 plasmid for SelW was constructed by Inovogen Tech. Co., and the gRNA sequences for SelW are shown in Table 1. The jetPEI™ (Polyplus-transfection Inc. USA) was used to deliver plasmids into chicken embryo and used nitrogen/phosphate (N/P) ratio of 8 to calculate amount of JetPEI required, as adopted by Jordan [40]. Chicken embryos at Stage X of development [41] were selected, and a 2-3 μL mixture of plasmid and jetPEI™ was introduced beneath the blastoderm. One week following the introduction of the mixture, muscles were frozen immediately in liquid nitrogen, and stored at −80°C until required. The DNA sequence of SelW was sequenced by Huada Gene Co., and the knockdown efficiency of SelW was detected by western blot technology.

**Table 1:** Sequences for Cas9 gRNA

	Sequence (5′-3′)
gRNA1	CTGCTTCCAGAACCCGCGCA
gRNA2	CCGGTGCCGTGCGCGGGTTC
gRNA3	CCAGAGCTCGCGTTCCAACC
gRNA4	GCCAAACTGCAGCGCATCG

### Quantitative real-time PCR (qPCR) analysis of mRNA levels

Total RNA was isolated [2] from muscles, myoblasts, and embryos using TRIzol reagent according to the manufacturer's instructions (Invitrogen, Shanghai, China). The RNA preparation, qPCR, and relative mRNA abundance quantification procedures were the same as previously described [16]. The amplification efficiency for each gene was determined using the DART-PCR program [42]. The relative abundance of mRNA was also calculated [43] to account for gene-specific efficiencies and was normalized to the mean expression of GAPDH and β-actin.

Primer Premier Software (Premier Biosoft International, USA) was used to design specific primers based on known chicken sequences (Table 2).

**Table 2:** Primers used in the present study

Genes	Forward primer (5′-3′)	Reverse primer (5′-3′)
RyR1	AGCCGAGCGTGGTCTATTAC	GAGGCAGTTGTAGCCGATGA
RyR3	TGGTTGAGGTAATGGCAGAA	TCTCCTTGGCTGTGAGTGTG
SERCA1s	CCCTGGTCACAACTCTGCTG	GTCAGTGGAACCTTGGCTGT
SLC8A1	ACCGCTTCATGTCCTCCA	ATGAGCCCAAAGCCATCA
PMCA1	GATGGAAGGCTCTGGAAGG	TTCAGTGGCTGCATTTCC
CACNA1S	CGAGGCCATGCTCAAGAT	CCAGGGAAACGATGGAGA
TRPC1	CGCTACCTCCACCTTTCAAT	CGTTTCACTTTGCCACTCG
TRPC3	GCAATCAGCAAGGGCTACAT	GTGCCGTCTTCGTCATAGG
STIM1	CGGCTTCCAGATCGTCAA	CATCCAGGTCATCCACGTC
ORAI1	GAGGTGGTGTTGCTGTGTTG	CTGCCTGTCCTGATGTGATG
β-actin	CCGCTCTATGAAGGCTACGC	CTCTCG GCTGTGGTGGTGAA
GAPDH	AGAACATCATCCCAGCGT	AGCCTTCACTACCCTCTTG

### Western blot analysis

Protein extracts from the muscles, myoblasts, and embryos were subjected to SDS-polyacrylamide gel electrophoresis under reducing conditions. Separated proteins were then transferred to nitrocellulose membranes using a tank transfer for 2 h at 200 mA in Tris-glycine buffer containing 20% methanol. The membranes were blocked with 5% skim milk for 2 h, and then incubated over night with diluted primary antibodies against SERCA (1:1000, Immunoway Biotechnology Company, USA) and SelW (made by our lab) followed by a horseradish peroxidase (HRP)-conjugated secondary antibody against rabbit IgG (1:3000, Santa Cruz Biotechnology, USA). To verify equivalent loading of samples, the membrane was incubated with monoclonal β-actin antibody (1:1000, Santa Cruz Biotechnology, USA), followed by an HRP-conjugated goat anti-mouse IgG (1:3000). The signal was detected using an enhanced chemiluminescence system (Cheml Scope5300, Clinx Science Instruments, Shanghai, China).

### Histological analysis of muscles

Histological analysis was performed according to our previous study [44]. After necropsy, the muscle tissue specimens were rapidly fixed in 10% neutral buffered formalin solution for at least 24 h. Fixed specimens were processed using the conventional paraffin-embedding technique. From the prepared paraffin blocks, sections were obtained and stained with hematoxylin and eosin (HE) for light microscopic examination.

### Determination of Ca^2+^ levels in chicken muscles

To determine the levels of Ca^2+^ in the chicken muscles, inductively coupled plasma mass spectrometry ICP-MS (Thermo iCAPQ, American) was used. The instrumental parameters of the equipment used are summarized in Table 3.

**Table 3:** Instrumental parameters for the ICP-MS

Items	Parameters
Frequency (MHz)	27.12
Reflect power (kW)	1.55
Sampling depth (mm)	5.0
Torch-H (mm)	0.01
Torch-V (mm)	−0.39
Carrier gas (L/min)	1.05
Nebuliser pump (rpm)	40
S/C temperature (°C)	2.7
Oxide ions (156/140)	<2.0%
Doubly charged (70/140)	<3.0%
Nebuliser type	Concentric

The Ca^2+^ concentrations were determined in by an acid digest of samples according to the method of Uluozlu et al. [45]. One gram of each sample was digested with 5 mL HNO_3_ (65%) and 2 mL H_2_O_2_ (30%) in a microwave digestion system and diluted to 10 mL with deionized water. A blank digest was carried out in the same way. All sample solutions were clear. Digestion conditions for the microwave system were applied as 1800 W for 3 min at 100°C, 1800 W for 10 min at 150°C, and 1800 W for 45 min at 180°C. All digested samples were filled with ultra-pure water to the final volume before analysis by ICP-MS.

### Ca^2+^ image in muscle tissue with SRμ-XRF

Ca^2+^ localization in muscle tissues were surveyed with synchrotron radiation micro X-ray fluorescence (SRμ-XRF). Muscle tissue specimens were rapidly fixed in 10% neutral-buffered formalin solution for at least 24 h. Fixed specimens were dehydrated through a graded series of ethanol, cleared in xylene, embedded in paraffin, and then cut into 5 mm-thick sections. The slices were fixed onto a 1 mm-thick glass slide, and then analyzed by SRμ-XRF according to the method of Li [46].

### Determination of Ca^2+^ level in cells

The cells were cultured in 35 mm glass-bottomed dishes and loaded for 30-60 min (37°C) with three different Ca^2+^ fluorescence indicators: 5 μM Fluo3 acetoxymethylester (AM) (Beyotime Institute of Biotechnology, Haimen, China) for the cytoplasm, 10 μM Rhod2 (Invitrogen) for the mitochondria [47], and 5 μM Fluo5N AM (Invitrogen) for the SR [48–50]. Rhod2 has a net positive charge, which promotes preferential sequestration in the mitochondria due to potential-driven uptake, whereas Fluo5N promotes dye accumulation within the SR [49]. To remove the cytosolic Rhod2 and Fluo 5N, the cells were permeabilized with 10 μM digitonin according to the method described previously [47]. To estimate the Rhod2 fluorescence pattern in live mitochondria, Mito-Tracker Green (Beyotime Institute of Biotechnology, Haimen, China) was used for mitochondrial marking [51]. In addition, we used ER-Tracker Red (Beyotime Institute of Biotechnology, Haimen, China) to mark SR [52]. Cells were washed three times and left in Tyrode's solution (in mM: 140 NaCl, 5.4 KCl, 1.8 CaCl_2_, 0.5 MgCl_2_, 10 Hepes, and 5.6 glucose, pH = 7.4) for 10 min until the cell fluorescence equilibrated. Images were acquired using excitation wavelengths of 488 nm for Fluo3, Fluo5N, and Mito-Tracker Green and 563-587 nm for Rhod2 and ER-Tracker Red. The signals were collected at 505-530 nm for Fluo3, Fluo5N and Mito Tracker Green and at 590 nm for Rhod2 and ER-Tracker Red. After the cell fluorescence was equilibrated and stabilized, Ni, ryanodine, Cd, caffeine, and thapsigargin were added to the cells, and the fluorescence was collected and imaged with a confocal laser scanning microscope, (Fluoview^TM^ FV 1000) using a 40× oil lens, and analyzed using the Olympus Fluoview Ver. 2.0a Viewer software. The fluorescence intensity levels are presented relative to baseline and shown as F/F_0_, where F_0_ is the initial fluorescence as described previously [53]. Fluorescence data were collected from an average of 8-12 cells per experiment.

### Determination of Ca^2+^ level in embryo skeletal muscle

Embryo muscle samples were homogenized on ice in physiological saline and centrifuged at 700 × *g* to collect supernatants. Ca^2+^ levels were determined using the assay kits (Nanjing Jiancheng Bioengineering Institute). Protein levels were determined using the protein assay kit (Nanjing Jiancheng Bioengineering Institute). The biochemical assays were performed according to the manufacturer's instructions with a UV-visible spectrophotometer (T6 Xinyue, Beijing).

### Antioxidative enzyme activity and oxidative injuries assays

Chicken and embryo muscle samples were homogenized on ice in physiological saline and centrifuged at 700× *g* to collect supernatants for the biochemical assays. GPx, SOD, and CAT activities; MDA concentration; H_2_O_2_ levels; and protein concentrations were determined according to the kit's instructions (Nanjing Jiancheng Bioengineering Institute, China) and were recorded with Multimode Plate Readers (TECAN Infinite M200 PRO, Switzerland).

### Detection of mitochondrial membrane potential in myoblasts

The mitochondrial membrane potential was monitored using 5, 5′, 6, 6′- tetra-chloro-1, 1′, 3, 3′-tetraethylbenzimidazolyl-carbocyanine iodide (JC-1), a lipophilic cationic fluorescence dye (Beyotime Institute of Biotechnology, Haimen, China) [54, 55]. Cells were incubated with the JC-1 staining solution at 37°C for 20 min and rinsed twice with JC-1 buffer. The mitochondrial membrane potentials were monitored by determining the relative amounts of the dual emissions from the mitochondrial JC-1 monomers and polymers using an Olympus fluorescence microscope and a Multimode Plate Reader (TECAN Infinite M200 PRO, Switzerland). The JC-1 monomer has an excitation wavelength of 490 nm and emission wavelength of 530 nm. The JC-1 polymer has an excitation wavelength of 525 nm and emission wavelength of 590 nm. With low mitochondrial membrane potential, JC-1 existed mainly as a monomer, showing a green fluorescence. JC-1 forms aggregate and emit red fluorescence whenever the mitochondrial membrane potential was high. The mitochondrial membrane potential was indicated by the green to red fluorescence intensity ratio.

### Sections for electron microscopy

The technique adopted to observe ultrastructural changes was similar to that of our previous study [44]: the collected samples were fixed immediately in 2.5% glutaraldehyde in phosphate-buffered saline (v/v, pH 7.2), post-fixed in 1% osmium tetroxide (v/v), and stained with 4.8% uranyl acetate following dehydration. The samples were washed in propylene oxide and impregnated with epoxy resins. The semi-fine sections were contrasted with uranyl acetate and lead citrate for study *via* microscopy. The microphotographs were taken with a transmission electron microscope (TEM).

### Statistical analysis

The data analysis was performed using SPSS statistical software for Windows (version13; SPSS Inc., Chicago, IL, USA). Differences between different groups were assessed using Student's *t*-test or one-way ANOVA. The data are expressed as the means ± standard deviation. Differences were considered to be significant at *P* < 0.05.
